# Large field-of-view scanning small-angle X-ray scattering of mammalian cells

**DOI:** 10.1107/S1600577520006864

**Published:** 2020-06-16

**Authors:** Chiara Cassini, Andrew Wittmeier, Gerrit Brehm, Manuela Denz, Manfred Burghammer, Sarah Köster

**Affiliations:** a Institute for X-ray Physics, University of Göttingen, Friedrich-Hund-Platz 1, 37077 Göttingen, Germany; b Cluster of Excellence ‘Multiscale Bioimaging: from Molecular Machines to Networks of Excitable Cells (MBExC)’, University of Göttingen, Germany; c European Synchrotron Radiation Facility, 71 Avenue des Martyrs, 38043 Grenoble, France

**Keywords:** biological cells, nanostructures, high throughput, scanning SAXS, image segmentation

## Abstract

X-ray imaging is a complementary method to electron and fluorescence microscopy for studying biological cells. In particular, scanning small-angle X-ray scattering provides overview images of whole cells in real space as well as local, high-resolution reciprocal space information, rendering it suitable to investigate subcellular nanostructures in unsliced cells. One persisting challenge in cell studies is achieving high throughput in reasonable times. To this end, a fast scanning mode is used to image hundreds of cells in a single scan. A way of dealing with the vast amount of data thus collected is suggested, including a segmentation procedure and three complementary kinds of analysis, *i.e.* characterization of the cell population as a whole, of single cells and of different parts of the same cell. The results show that short exposure times, which enable faster scans and reduce radiation damage, still yield information in agreement with longer exposure times.

## Abstract 2

A fast-scanning small-angle X-ray scattering study of biological cells is presented that achieves higher throughput and requires lower dose than previous experiments.

## 1. Introduction   

Imaging biological cells with a spatial resolution sufficient for identifying subcellular structures is a very challenging task, currently tackled mainly by three kinds of probes: electrons, visible-light fluorescence and X-rays. Electron microscopy (Koster & Klumperman, 2003 ▸; de Jonge *et al.*, 2009 ▸) yields the best spatial resolution, resolving details down to the subnanometer range. However, it requires extensive sample preparation, typically including slicing and staining of the sample. Thanks to super-resolution techniques (Hell, 2007 ▸), fluorescence microscopy is widely used in labeled, intact cells (Fernández-Suárez & Ting, 2008 ▸; Huang *et al.*, 2010 ▸) and can resolve details on the order of tens of nanometers. X-ray imaging techniques (Kirz *et al.*, 1995 ▸; Hémonnot & Köster, 2017 ▸) rely on the small wavelength and high penetration depth of X-radiation. In particular, scanning small-angle X-ray scattering (SAXS) (Fratzl *et al.*, 1997 ▸) is used on unsliced, unstained samples to obtain both real and reciprocal space information. A large variety of samples can be examined with this technique, including, but not limited to, bone (Fratzl *et al.*, 1997 ▸; Bünger *et al.*, 2006 ▸; Gourrier *et al.*, 2010 ▸; Turunen *et al.*, 2014 ▸), wood (Fratzl *et al.*, 1997 ▸; Lichtenegger *et al.*, 1999 ▸) and teeth (Kinney *et al.*, 2001 ▸; Gaiser *et al.*, 2012 ▸). In real space, the dark-field contrast image (Bunk *et al.*, 2009 ▸) offers an overview of the scanned area. The real-space resolution is limited by the dimensions of the X-ray beam and the step size of the scan. In reciprocal space, scanning SAXS can access the nanometer range *via* scattering patterns collected at each position of the scan. Thus, moderate resolution in real space is complemented by high resolution in reciprocal space. Thanks to this unique combination, several subcellular structures were studied in whole cells, including keratin bundles in SK8/18-2 cells (Weinhausen *et al.*, 2012 ▸, 2014 ▸; Hémonnot *et al.*, 2016*a* ▸), actin bundles in hair cell stereocilia (Piazza *et al.*, 2014 ▸) and in *Dictyostelium discoideum* (Priebe *et al.*, 2014 ▸) and chromatin in 3T3 fibroblasts (Hémonnot *et al.*, 2016*b* ▸). A model-free diffraction pattern analysis was demonstrated for several cell types (Bernhardt *et al.*, 2016 ▸). Notably, all these studies typically took into account only about 2–30 cells in total; when different cell types (Bernhardt *et al.*, 2016 ▸), differently prepared samples (Weinhausen *et al.*, 2014 ▸; Priebe *et al.*, 2014 ▸) or cells in different stages of the cell cycle (Hémonnot *et al.*, 2016*b* ▸) were compared, each of the compared groups included at most ten cells.

Since cell-to-cell variability occurs even within the same monoclonal population (Pelkmans, 2012 ▸), any cellular assay should include a statistically significant number of cells. However, achieving high resolution for a large number of cells in a single experiment is very challenging. The conditions for high spatial resolution usually limit the accessible field-of-view. This results in a low number of cells per acquisition, thus longer times are needed for large numbers of cells to be assessed. Recent attempts to overcome these limitations include, for super-resolution fluorescence techniques, the development of a large and uniform epi-illumination (Douglass *et al.*, 2016 ▸) or the decoupling of illumination and detection pathways with slab waveguides (Diekmann *et al.*, 2017 ▸). Multiple electron beams can extend the field-of-view of scanning electron microscopy by simultaneously scanning as many regions as the number of beams employed (Eberle *et al.*, 2015 ▸). Field-of-view expansion has been demonstrated for scanning SAXS on cardiac tissue, thanks to a novel fast scanning mode (Nicolas *et al.*, 2017 ▸) that resulted in a field-of-view of 6 mm × 5 mm with a pixel size of 5 µm for the dark-field contrast image.

Here, we use fast scanning SAXS to study single cells, thus accessing the subcellular structural information provided by the scattering patterns, corresponding to typical lengths of a few nanometers to a few tens of nanometers, while obtaining a panoramic view of the entire cell population (1.5 mm × 1.5 mm field-of-view) from the dark-field contrast image. Regardless of the imaging method, measurements carried out with high resolution over a large field-of-view lead to vast amounts of data that need to be handled in a time-efficient manner (Eberle *et al.*, 2015 ▸; Nicolas *et al.*, 2017 ▸; Beghin *et al.*, 2017 ▸). Here, we present a segmentation strategy for the dark-field contrast image that is key to dividing the data into regions of interest (ROIs), thus enabling further analysis in feasible times. Different levels of analysis, focusing on the overall cell population, on the characteristics of different cells or on the properties of different parts of the same cell are illustrated, as well as an application example that corroborates previous results with data from a more substantial number of cells. In particular, we pay attention to effects of radiation damage, which still poses a major challenge when imaging biological matter by X-rays. We thus enable statistically meaningful data acquisition and analysis by scanning SAXS and render it a complementary method to other nanoscale imaging methods.

## 2. Materials and methods   

### 2.1. Cell culture and sample preparation   

NIH-3T3 fibroblasts derived from Swiss albino mouse embryos (Todaro & Green, 1963 ▸) were cultured in high glucose (4.5 g L^−1^) Dulbecco’s Modified Eagle’s Medium (DMEM, D6429; Sigma-Aldrich, Merck KGaA, Darmstadt, Germany) supplemented with 10% (*v*/*v*) fetal bovine serum (F0804; Sigma-Aldrich), 100 units mL^−1^ penicillin and 0.1 g L^−1^ streptomycin. The culture flasks were kept in a cell incubator at 37°C in a water-saturated atmosphere with 5% CO_2_. The cells were transferred onto the flat side of Si_3_N_4_ membranes (frame size 5 mm × 5 mm, window size 1.5 mm × 1.5 mm, membrane thickness 1000 nm; Silson Ltd, Warwickshire, UK) when they reach 80% confluence, by detaching them from the culture flasks using 0.05% (*v*/*v*) trypsin (T4799-5G; Sigma-Aldrich) and 0.02% (*w*/*v*) EDTA (8040.2; Carl Roth GmbH, Karlsruhe, Germany) in phosphate buffered saline (PBS). The membranes were seeded with an initial concentration of about 3.8 × 10^8^ cells mL^−1^. After about 24 h, the windows were washed with PBS, fixed (Hémonnot *et al.*, 2016*b* ▸) for 15 min with 3.7% formaldehyde solution stabilized with 1% methanol (104003; Merck, diluted 1:10 in PBS) and then washed three times with PBS. Fixed samples were washed in ultrapure water and plunge-frozen (Weinhausen *et al.*, 2012 ▸; Priebe *et al.*, 2014 ▸; Bernhardt *et al.*, 2016 ▸; Hémonnot *et al.*, 2016*b* ▸) by fast immersion in a liquid ethane–propane mixture using an automatic grid plunger (EM GP2; Leica Microsystems GmbH, Wetzlar, Germany). The frozen samples were then lyophilized in a home-built freeze-drier (Weinhausen *et al.*, 2012 ▸; Priebe *et al.*, 2014 ▸; Bernhardt *et al.*, 2016 ▸; Hémonnot *et al.*, 2016*b* ▸). Visible-light phase contrast imaging was carried out before, between and after these steps for quality control.

### 2.2. Scanning SAXS   

We performed scanning SAXS experiments at the micro-branch (experimental hutch II) of beamline ID13 at the European Synchrotron Radiation Facility (ESRF, Grenoble, France). The beam was pre-focused by parabolic beryllium compound refractive lenses (Be-CRLs) of 200 µm radius at the apex (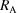) and monochromated by a Si-111 channel-cut monochromator to a photon energy of 13.0 keV. The beam was then focused by Be-CRLs with an 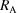 of 50 µm to 2 µm × 3 µm spot size and a flux of 1.7 × 10^12^ photons s^−1^. Close to the sample, the beam was conditioned with a 20 µm aperture and cleaned with an 80 µm guard aperture (pinhole camera). The sample was aligned with an on-axis visible-light microscope. Downstream of the sample, a 70 mm helium-filled flight tube was employed to reduce air scattering. A beam stop right outside the exit window of the flight tube blocked the primary beam, while the scattered radiation was recorded by an Eiger X 4M detector (2070 rows × 2167 columns, *i.e.* ∼4 megapixels, pixel size 75 µm × 75 µm; Dectris, Baden, Switzerland), located about 0.9 m away from the sample. A fast scanning mode (Nicolas *et al.*, 2017 ▸) was achieved by continuously moving the sample at constant speed during data acquisition.

**Figure 1:**
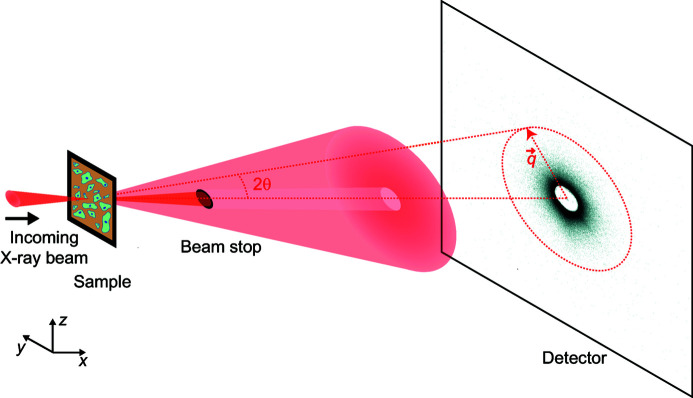
Experimental setup. A Si_3_N_4_ membrane with freeze-dried cells is moved along the *y*- and *z*-axes through the X-ray beam to obtain a raster scan. The undiffracted beam is blocked by the beam stop; the scattered X-rays are collected by the detector. The red dashed lines illustrate the relationship between the scattering angle 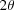 and the direction of the corresponding scattering vector 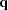.

We used the fast scanning mode on the freeze-dried NIH-3T3 fibroblasts grown on Si_3_N_4_ windows. Each window contained about 800 cells on a 1.5 mm × 1.5 mm area. Scans of a window were obtained by moving the window horizontally (2974 positions) and vertically (2991 positions) through the X-ray beam (Fig. 1 ▸) in steps of 0.5 µm. These window-wide scans were performed using the minimum exposure time allowed by the detector, *i.e.* 1.34 ms per scan position. This way, each scan consisted of 8895234 scattering patterns in total, acquired in about 7 h (25602 s, including about 1.54 ms overhead per scan position). For comparison, we also performed scans of smaller regions containing single cells with longer exposure times (20 ms per scan position), comparable with cell scans performed in the past (Weinhausen *et al.*, 2014 ▸; Hémonnot *et al.*, 2016*a* ▸,*b* ▸). For both short and long exposure time scans, the step size was 0.5 µm × 0.5 µm. The radiation dose *D* can be estimated as shown by Weinhausen *et al.* (2012 ▸) and Hémonnot *et al.* (2016*b* ▸) following Howells *et al.* (2009 ▸), who approximate the cellular material with an ‘average protein’ of empirical formula H_50_C_30_N_9_O_10_S. Accordingly, we used the equation
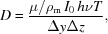
where 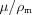 = 2.55 cm^2^ g^−1^ is the ratio between mass attenuation coefficient and mass density of the cellular material (Berger *et al.*, 2010 ▸), 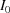 is the photon flux, 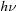 is the photon energy, *T* is the exposure time per scan point and 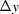 and 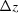 are the step sizes of the scan in the horizontal and vertical direction, respectively. The resulting doses were 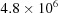
 Gy for 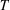 = 1.34 ms and 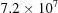
 Gy for 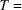 20 ms.

### 2.3. Data analysis   

For each scan, a dark-field contrast image (Bunk *et al.*, 2009 ▸; Weinhausen *et al.*, 2012 ▸, 2014 ▸; Priebe *et al.*, 2014 ▸; Hémonnot *et al.*, 2016*b* ▸) of the scanned region was obtained by integrating the 2D scattering patterns within a region of interest on the detector corresponding to a maximum *q* value of 2.99 nm^−1^ and by plotting the resulting intensity values in a color-coded fashion at the corresponding scan positions. *q* is the magnitude of the scattering vector 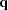,
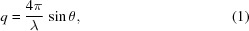
where λ is the wavelength of the incoming X-rays and θ is half the scattering angle (see Fig. 1 ▸).

**Figure 2:**
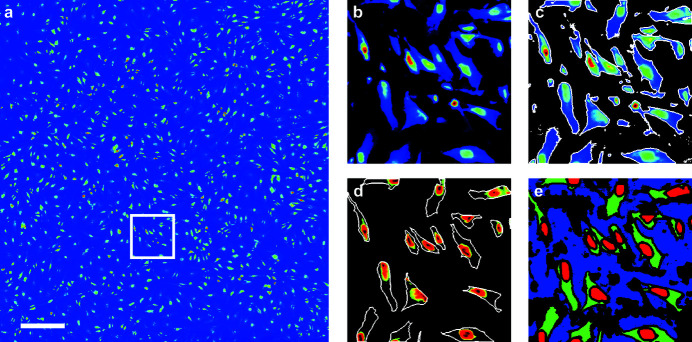
Dark-field contrast image segmentation. (*a*) X-ray dark-field contrast image for the complete scan of the entire window. Scale bar: 200 µm. (*b*) Detail of the region inside the white box in (*a*). (*c*) Result of local thresholding, showing the same region as in (*b*), but the pixels identified as background have been masked out. The contours of the mask are shown in white. (*d*) Result of global thresholding on single cells, showing the same region as in (*b*), but only the nuclei. The contours of the cell bodies are shown in white. (*e*) Final regions of interest for the region shown in (*b*). Background is shown in blue, cytoplasm in green and nuclei in red. Black pixels are disregarded. The color scales have been readjusted individually in (*a*)–(*d*) for better visualization.

In order to define ROIs for our large datasets, we segmented the corresponding dark-field contrast image, where we could distinguish the cells from the background and the nuclei from the cytoplasm, as exemplified in Figs. 2(*a*) and 2(*b*) ▸. The large number of cells grown on one window renders manual ROI selection unreasonable. Therefore, we used a semi-automated segmentation procedure detailed in the supporting information. In brief, we used local intensity thresholds (Bradley & Roth, 2007 ▸) to separate the cells from the background [Fig. 2(*c*) ▸] and a different Otsu threshold (Otsu, 1979 ▸) for each cell to find the nuclei [Fig. 2(*d*) ▸]. The final ROIs are shown in Fig. 2(*e*) ▸ for the portion of the dark-field contrast image in the white box in Fig. 2(*a*) ▸ and in Fig. 4(*a*) for the complete frame.

One-dimensional radial intensity profiles 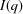 were obtained from the 2D scattering patterns by azimuthal integration (Bunk *et al.*, 2009 ▸; Weinhausen *et al.*, 2012 ▸, 2014 ▸; Priebe *et al.*, 2014 ▸; Hémonnot *et al.*, 2016*a* ▸) in the same *q* range as used for the dark-field contrast image computation and are represented as a function of the scattering vector magnitude *q* [equation (1)]. The radial intensity profiles were then normalized by the exposure time and the background intensity was subtracted from the nuclear and cytoplasmic intensities. The background-corrected radial intensity curves were fitted by a non-linear least-squares minimization to a power law in the *q* range [0.185, 1.723] nm^−1^, corresponding to real-space features between 3.6 nm and 34.0 nm.

All data analysis was carried out using self written MATLAB R2017b (The MathWorks, Inc., Natick, MA, USA) scripts, including the Image Processing Toolbox and functions from the Nanodiffraction toolbox developed by Nicolas *et al.* (2017 ▸).

## 3. Results and discussion   

### 3.1. Analysis of cell populations, single cells and subcellular positions   

**Figure 3:**
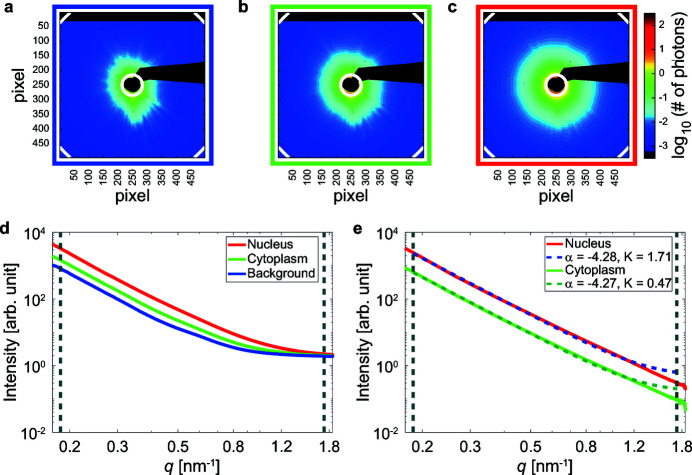
Data reduction and fitting. (*a*) Average scattering pattern for the background region. (*b*) Average scattering pattern for the cytoplasmic region. (*c*) Average scattering pattern for the nuclear region. The white lines in (*a*)–(*c*) delimit the fitting range [see (*e*)]. (*d*) Radial intensity profiles for the background, cytoplasmic and nuclear regions, respectively, obtained by azimuthal integration of the average scattering pattern shown in (*a*), (*b*), (*c*). The vertical dashed lines delimit the fitting range [see (*e*)]. (*e*) Radial intensity profiles for the cytoplasmic and nuclear regions after background subtraction, fitted with equation (2). The vertical dashed lines delimit the fitting range.

The segmentation of the dark-field contrast image described above is used to compute the average scattering pattern for each ROI [Figs. 3(*a*), 3(*b*) and 3(*c*) ▸]. Each ROI consists of a large number of scattering patterns — 3610683 for the background, 700766 for the cytoplasm and 419836 for the nuclei — and possible anisotropies due to local orientations are not visible in these average scattering patterns. Indeed, all anisotropies stem from the background scattering pattern, as confirmed by background scattering pattern subtraction (see the supporting information). Thus, the isotropy of the averaged patterns justifies the computation of one-dimensional radial intensity profiles through azimuthal integration of the two-dimensional scattering patterns. The resulting intensity values *I* are plotted in Fig. 3(*d*) ▸ against *q* [see equation (1)]. The background radial intensity profile [blue curve in Fig. 3(*d*) ▸] is then subtracted from the two curves containing the actual signal [red and green curves in Fig. 3(*d*) ▸] to obtain the background-corrected radial intensity profiles shown in Fig. 3(*e*) ▸. These profiles follow a power law decay,
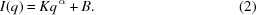
The additive constant *B* accounts for small density fluctuations in the sample, inelastic and incoherent scattering (Ruland, 1971 ▸; Gourrier *et al.*, 2010 ▸). The exponent α is related to the morphology of the sample. For instance, monodisperse rods would lead to α = −1, monodisperse platelets to α = −2 and monodisperse spheres to α = −4 (Porod, 1951 ▸; Guinier & Fournet, 1955 ▸). Non-integer exponents can be caused by polydisperse and/or fractal scatterers (Koberstein *et al.*, 1980 ▸; Schmidt, 1982 ▸; Martin & Hurd, 1987 ▸). In particular, fractals are characterized by α > −4 and values <−4 indicate polydispersity and heterogeneity. In the latter case, predictions on the precise value of α can only be made if a model for the electron density distribution is assumed. When α = −4 at large *q* values, equation (2) is Porod’s law (Porod, 1951 ▸; Guinier & Fournet, 1955 ▸). In this case, Porod’s constant *K* depends on the electron density of the sample and the surface area of the interface between scatterers and air (Porod, 1951 ▸; Guinier & Fournet, 1955 ▸).

**Figure 4:**
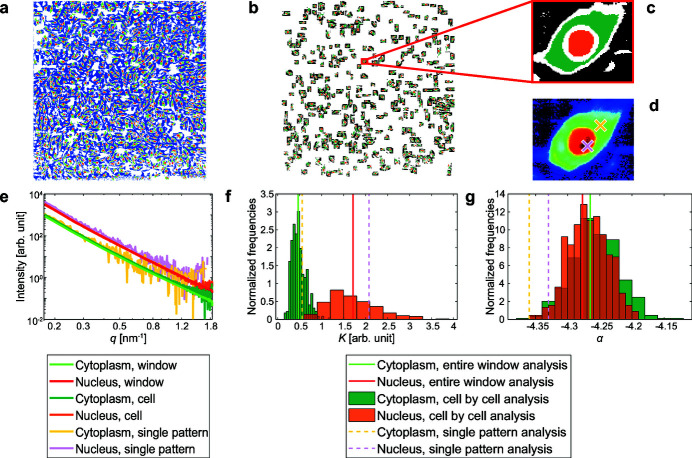
Analysis of different data ensembles. (*a*) ROIs, entire window analysis. Background is shown in blue, cytoplasm in green and nuclei in red. (*b*) ROIs, cell-by-cell analysis. Background is shown in black, cytoplasm in teal and nucleus in orange. (*c*) Enlargement of the region in the red box in (*b*): example of ROIs for one cell. (*d*) Dark-field contrast image for the cell shown in (*c*). The positions of the scattering patterns used to exemplify single scattering pattern analysis [see (*e*), (*f*), (*g*)] are marked by crosses in yellow for the cytoplasm and pink for the nucleus. (*e*) Radial intensity profiles for the cytoplasmic and nuclear regions, after background subtraction. The curves were obtained by azimuthal integration of average scattering patterns coming from different ensembles: averaged over the entire window [ROIs shown in (*a*)], averaged over an individual cell [ROIs shown in (*c*)] or single (not averaged) scattering patterns [marked in *d*)]. (*f*) Distribution of *K* values obtained from the cell subset shown in (*b*). The values obtained from the entire window analysis and from the single scattering pattern analysis are also shown. (*g*) Distribution of α values obtained from the cell subset shown in (*b*). The values obtained from the entire window analysis and from the single scattering pattern analysis are also shown. For each bin in (*f*) and (*g*), the frequencies are obtained by dividing the counts by the total number of values and then normalized by dividing them by the width of the bin.

The same analysis can be repeated on ensembles of scattering patterns, allowing us to compare different cells, or groups of cells. By accessing one cell at a time, we can compute background, cytoplasmic and nuclear average scattering patterns for each cell. For example, for the ROIs shown in Fig. 4(*c*) ▸, we obtain the radial intensities shown in Fig. 4(*e*) ▸ (teal curve for the cytoplasm, orange curve for the nucleus). These curves are very similar to those obtained from averaging over the entire window [ROIs shown in Fig. 4(*a*) ▸], also plotted in Fig. 4(*e*) ▸ for comparison (light green curve for the cytoplasm, red curve for the nucleus). The main difference is that the single cell curves are slightly noisier for high *q* values, which is not surprising since they were obtained by averaging a much smaller number of scattering patterns, *i.e.* 1373 instead of 700766 for the cytoplasm and 461 instead of 419836 for the nucleus. It is now possible to select a subpopulation of cells, for instance imposing conditions on the cell size. An example is shown in Fig. 4(*b*) ▸, where only the ROIs belonging to the cells satisfying 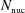 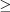 30 , 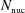 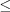 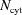 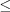 5000 and 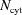 < 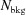 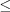 10000 are shown (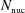, 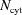 and 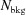 are the numbers of pixels included in the nuclear, cytoplasmic and background region, respectively). We empirically set these conditions to reduce the number of connected components that are fragments of cells instead of whole cells, while retaining a statistically significant number of cells. For further details, see the supporting information. *K* and α are analyzed with respect to this subpopulation by computing the average nuclear, cytoplasmic and background scattering patterns for each of the cells shown in Fig. 4(*b*) ▸, by plotting the corresponding radial intensities and fitting them with equation (2). The result is a set of fit parameters, in particular *K* and α, for each of the 444 analyzed cells. The corresponding distributions are shown in Figs. 4(*f*) and 4(*g*) ▸. For comparison, the *K* and α values obtained for the entire window are depicted as vertical solid lines. These values are close to the average values of the corresponding distributions (see also the supporting information, Table S2), suggesting that the subpopulation used here is representative of the total population. The highly overlapping distributions of α for nuclei (orange) and cytoplasm [teal; Fig. 4(*g*) ▸] suggest that all cells have a similar nanostructure, with little difference between nuclear and cytoplasmic regions. Differences between nuclei and cytoplasm emerge in the *K* distributions [Fig. 4(*f*) ▸] that have large standard deviations (as shown in Table S2 in the supporting information), reflecting the high variability occurring even among the same cell line.

The radial intensities can also be evaluated for each scan point within the cell body separately, *i.e.* without any averaging, so that the local variability is accessed. In this case, the assumption of isotropic scattering patterns is valid in first approximation only. An example is shown for just two positions, indicated in Fig. 4(*d*) ▸ by a yellow cross (cytoplasm) and a pink cross (nucleus). The background is computed by averaging those background scattering patterns belonging to the surroundings of this cell [black pixels in Fig. 4(*c*) ▸] that are on the same row as the cytoplasmic or nuclear scattering pattern considered. Due to the considerable length of one row, scattering patterns in different rows are acquired at very distant time points and, as the incoming beam intensity can fluctuate in time, using only background scattering patterns acquired very soon before or after the considered scattering pattern ensures that the incident X-ray intensity levels are not significantly different for the considered scattering pattern and its background. The resulting background-subtracted radial intensities, shown in Fig. 4(*e*) ▸, follow the curves for the whole window well, but are visibly noisier than the others. Moreover, they show an increase at large *q* values, which is actually an artifact of the azimuthal integration procedure for low-intensity values (see the supporting information). However, the fits of these lower-quality data with a power law are still possible, and the resulting *K* and α values [vertical dashed lines in Figs. 4(*f*) and 4(*g*) ▸] fall within the boundaries of the distributions obtained for the single cell averages.

### 3.2. Validity of data from short exposure times   

The fits of the comparatively noisy individual radial intensity profiles shown in Fig. 4(*e*) ▸ yield results in good agreement with those obtained from averages over whole cells [see Figs. 4(*f*) and 4(*g*) ▸], suggesting that even these low-intensity, non-averaged single scattering patterns contain valuable information that can be analyzed. The analysis of non-averaged scattering patterns from scanning SAXS has already been successfully applied not only on strongly scattering materials such as bone (Fratzl *et al.*, 1997 ▸; Bünger *et al.*, 2006 ▸; Turunen *et al.*, 2014 ▸; Gourrier *et al.*, 2010 ▸), wood (Fratzl *et al.*, 1997 ▸; Lichtenegger *et al.*, 1999 ▸) or teeth (Kinney *et al.*, 2001 ▸; Gaiser *et al.*, 2012 ▸) but also on weakly scattering samples, such as biological cells (Weinhausen *et al.*, 2012 ▸, 2014 ▸; Bernhardt *et al.*, 2016 ▸; Hémonnot *et al.*, 2016*a* ▸,*b* ▸). However, in all previous examples, the exposure times ranged from 30 ms to 10 s per scan point, with typical doses (Howells *et al.*, 2009 ▸; Weinhausen *et al.*, 2012 ▸; Bernhardt *et al.*, 2016 ▸) of the order of 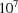
–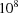
 Gy, thus being considerably longer and more invasive than here (1.34 ms, corresponding to a dose of 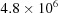
 Gy), and therefore preventing the recording of large data sets. The scattering patterns obtained with longer exposure times do not necessarily yield more information than those from shorter exposure times, as radiation damage plays an increasingly more important role when more dose is imposed on the sample (Leccia *et al.*, 2010 ▸; Kosior *et al.*, 2012 ▸; Gianoncelli *et al.*, 2015 ▸).

**Figure 5:**
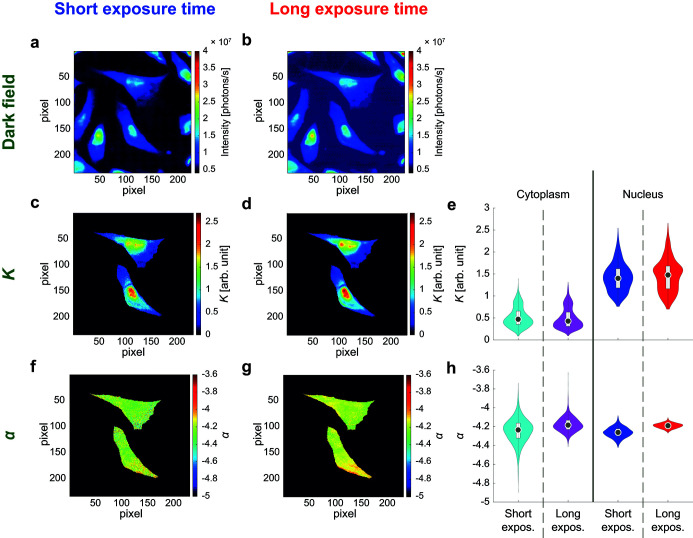
Comparison of fast and slow scans. (*a*) Dark-field contrast image of one particular region from a fast scan (exposure time: 1.34 ms). (*b*) Dark-field contrast image of the same region from a slow scan (exposure time: 20 ms). (*c*) Map of *K* values, fast scan and (*d*) slow scan. (*e*) Violin plots of the *K* values shown in (*c*,  *d*). (*f*) Map of α values, fast scan and (*g*) slow scan. (*h*) Violin plots of the α values shown in (*f*), (*g*). In the violin plots (Bechtold, 2016 ▸; Hintze & Nelson, 1998 ▸) in (*e*) and (*h*), the gray circles mark the median value and the white boxes represent the interquartile range.

We compare the results obtained from a specific region in our full window fast scans to slower scans of the identical cells, using the same step size but a different exposure time, *i.e.* 20 ms per scan point, corresponding to a dose of 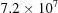
 Gy, added to the dose from the previous exposure, thus 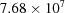
 Gy. This exposure time is chosen to maximize the signal-to-noise ratio (SNR) of the dark-field contrast image (for more details, see the supporting information). Fig. 5(*a*) ▸ shows a portion of the dark-field contrast image from Fig. 2(*a*) ▸, while Fig. 5(*b*) ▸ shows the dark-field contrast image for a scan, carried out later, on the same region, with 20 ms exposure time. Despite the intensities being normalized with respect to the different exposure times, the two images are not identical: the background appears brighter for the longer exposure time. This might be due to a change in the cell-to-substrate contrast caused by radiation damage, to artifacts introduced by the detector, to intensity fluctuations of the incoming X-ray beam, to undetected defective detector pixels randomly switching on and off, or to a combination of the factors above.

A single scattering pattern analysis is performed on the two central cells of the region, with the procedure described above, yielding the *K* and α maps in Figs. 5(*c*), 5(*d*), 5(*f*) and 5(*g*) ▸. The *K* maps look very similar to each other, although the one for the shorter exposure time [Fig. 5(*c*) ▸] appears more pixelated. Their similarity is supported by the strong agreement of the distributions of *K* for cytoplasmic and nuclear regions as shown in Fig. 5(*e*) ▸. The two distributions also have similar, although not identical, medians, standard deviations and averages, as reported in Table S3. The distributions of α resemble each other as well [Fig. 5(*h*) ▸] and their ranges strongly overlap, although the cytoplasmic distributions differ in their extreme values and the nuclear distribution for the long exposure time is shifted to larger values with respect to the distribution for the short exposure time. Of note, the α maps [Figs. 5(*f*) and 5(*g*) ▸] are fairly homogeneous, indicating a similar local morphology throughout the cell, in agreement with previous work (Hémonnot *et al.*, 2016*b* ▸). In fact, all four distributions shown in Fig. 5(*h*) ▸ have similar medians and averages, as can be seen in Table S3. At a closer look, these values are more similar within the same scan (*i.e.* between the different cellular regions) than within the same cellular region (*i.e.* nucleus or cytoplasm) in different scans. This suggests that α is determined by the exposure time rather than by the point in the cell that the signal originated from. Indeed, a previous study on the same cell line (Hémonnot *et al.*, 2016*b* ▸) suggests that evident differences among α values are related to a different severity of radiation damage. The authors show that freeze-dried samples lead to α ≃ −3.6, freeze-dried samples scanned with an attenuated beam intensity to α ≃ −4 and cryoprotected freeze-dried samples to α ≃ −4.3. Moreover, the sample preparation seems to influence the power law exponent values, as found for SK8/18-2 cells (Weinhausen *et al.*, 2014 ▸) and for *Dictyostelium discoideum* (Priebe *et al.*, 2014 ▸). Regardless of the cell type, living cells have larger exponents than chemically fixed cells, which in turn have larger exponents than frozen-hydrated cells, and freeze-dried cells yield the smallest exponents. It is possible that the low variation we observe for α throughout a cell is due to the freeze-drying procedure we apply. The aforementioned study of SK8/18-2 cells reports a larger difference between the average nuclear and cytoplasm exponents, for both living and hydrated cells, than what we measure here. However, it should be kept in mind that in this work we are dealing with a different cell line and with a single scattering pattern analysis rather than an average scattering pattern analysis.

In addition to radial intensity profiles the analysis of orientation and anisotropy of scattering patterns has recently been used on biological tissues and cells to obtain orientation maps for *Dictyostelium discoideum* (Priebe *et al.*, 2014 ▸), several types of human and murine cells (Bernhardt *et al.*, 2016 ▸), cardiomyocytes (Bernhardt *et al.*, 2017 ▸) and cardiac tissue (Nicolas *et al.*, 2017 ▸). We perform an analysis of orientation and anisotropy for the scattering patterns of the fast and slow scans shown in Fig. 5 ▸, following Bernhardt *et al.* (2016 ▸) and Nicolas *et al.* (2017 ▸), as explained in the supporting information, including Fig. S5. We find that, regardless of the exposure time and of the examined cell, a predominant orientation of about 21° emerges. This is an indication that the signal from the sample is not strong enough to allow for this kind of analysis. Indeed, orientations significantly different from 21° are only visible in some parts of the nuclei, where the signal is stronger, *i.e.* more scatterers are present in the beam, as the nucleus is thicker and denser than the cytoplasm. Analysis of orientation and anisotropy will strongly benefit from new-generation synchrotrons such as the current ESRF-EBS upgrade, as a higher brilliance will compensate for the faintness of cellular signals.

### 3.3. Dependence of the fit parameters on the cell size   

**Figure 6:**
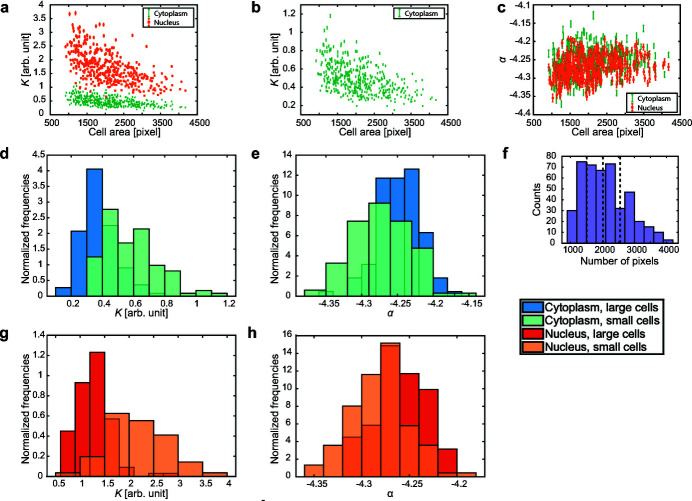
Dependence of *K* and α on the cell area for a subpopulation of cells. (*a*) Dependence of *K* and (*c*) α from the cell-by-cell analysis (see Fig. 4 ▸) on the cell size. (*b*) Same as (*a*), rescaled for the cytoplasm. (*d*) Distributions of the *K* and (*e*) α values for the cytoplasmic regions, for the cells in the first (light blue) and fourth (dark blue) area quartile. (*f*) Distribution of the cell areas for the subpopulation. The vertical dashed lines (corresponding to 1510 pixels, 2015 pixels and 2550 pixels) delimit the quartiles of the area distribution. (*g*) Distributions of the *K* and (*h*) α values for the nuclear regions, for the cells in the first (pale orange) and fourth (dark orange) area quartile. For each bin in (*d*), (*e*), (*g*) and (*h*), the frequencies are obtained by dividing the counts by the total number of values and then normalized by dividing them by the width of the bin.

The possibility to analyze one cell at a time allows us to compare various properties of a large number of cells. As an example, we show (Fig. 6 ▸) the dependence of *K* and α on the cell area, for the subpopulation shown in Fig. 4(*b*) ▸. For each cell, the cell area is quantified by counting the pixels belonging to the given cell, according to the cell body mask discussed above. Although there is no perfect anticorrelation, *K* evidently tends to decrease with increasing cell area [Fig. 6(*a*) ▸]. This is true for both the nucleus and the cytoplasm. Conversely, no dependence of α on the cell area emerges in Fig. 6(*c*) ▸.

The cell size is related to the phase of the cell cycle (Anderson *et al.*, 1969 ▸): the cellular volume grows during the gap 1 (G1) phase; the DNA is duplicated during the synthesis (S) phase; the cellular volume grows again during the gap 2 (G2) phase, then the cell divides (mitosis, M, and cytokinesis) into two ‘daughter’ cells that usually enter their own G1 phase (Alberts *et al.*, 2002 ▸). These volume changes appear in our two-dimensional dark-field contrast image of adherent cells [Fig. 2(*a*) ▸] as changes in the cell (projected) area. Since there is no one-to-one correspondence between cell size and phase of the cell cycle (Anderson *et al.*, 1969 ▸), it is not possible to tell the exact point of the cell cycle for a cell looking at its area only; nevertheless, among the smaller cells there is a higher incidence of cells in the G1/S phase, and among the bigger cells there is a higher incidence of cells in the G2/M phase (Hémonnot *et al.*, 2016*b* ▸). Therefore, we expect the cells in the first quartile of the area distribution [see Fig. 6(*f*) ▸] to be mostly in the G1/S phase and the cells in the fourth quartile to be mostly in the G2/M phase. For the sake of simplicity, in the following we refer to the cells in the first quartile of the area distribution as ‘small’ cells and to those in the fourth quartile as ‘large’ cells.

The distributions of α for small and large cells are globally different, as the two-sample Kolmogorov–Smirnov test (Darling, 1957 ▸) yields a *p*-value of 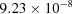 for the cytoplasmic distributions and of 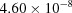 for the nuclear distributions. Both values are well below the commonly used 0.05 *p*-value threshold. However, only a very small shift to larger values is observed for the large cells, both for the cytoplasm [Fig. 6(*e*) ▸] and for the nucleus [Fig. 6(*h*) ▸]. The average values of these distributions are equal within experimental error (see also Table S4 in the supporting information). As already discussed, the exponent α is determined by the morphology of the sample. In the present case, the exponents are quite close to −4, which is the exponent associated with identical three-dimensional scatterers with well defined boundaries (Porod, 1951 ▸; Guinier & Fournet, 1955 ▸; Ruland, 1971 ▸; Koberstein *et al.*, 1980 ▸). However, all exponents we find are slightly but systematically smaller than −4, thus hinting to three-dimensional, heterogeneous scatterers, possibly having diffuse boundaries. Additional information is provided by *K*, that displays a much more evident difference in the distributions for small and large cells, both for the cytoplasm [Fig. 6(*d*) ▸] and for the nucleus [Fig. 6(*g*) ▸]. The distributions for the small cells are wider, *i.e.* they have larger standard deviations (see also Table S4 in the supporting information) and are centered on larger values. The difference between the average values is evident (as can be seen in the supporting information, Table S4), and the two-sample Kolmogorov–Smirnov test produces extremely low values (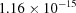 for the cytoplasmic *K* distributions and 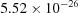 for the nuclear *K* distributions). This suggests a decrease of *K* from the earlier to the later phases of the cell cycle. When α = −4 and the scatterers have a uniform electron density, *K* coincides with Porod’s constant, which is proportional to the square of the electron density contrast 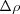 and to the surface area *S* of the interface between scatterers and air (Porod, 1951 ▸; Guinier & Fournet, 1955 ▸),
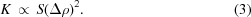
Assuming this holds for our data in first approximation, it follows that, during the cell cycle, there is a decrease in 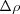, or in *S*, or both. As we perform our measurements in air, changes in the electron density contrast 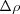 are equivalent to changes in the electron density of the scatterers, which, in turn, is directly proportional to the mass density of the scatterers. We speculate that a decrease in mass density could be caused by a rearrangement of proteins that are expressed anew during the growth phases of the cell cycle into their final conformation. A decrease of *S* is supposedly due to scatterers being packed in a different manner, and thus with a smaller exposed surface, in later phases of the cell cycle compared with the earlier phases.

## 4. Conclusions   

We demonstrate that fast scanning SAXS experiments on a large number of mammalian cells are possible, thanks to the synchronization of the continuous movement of the sample stage with the data acquisition (Nicolas *et al.*, 2017 ▸). This approach brings scanning SAXS to an entirely new level as it is now possible to acquire and analyze data from reasonably large populations of cells so as to draw statistically valid conclusions, despite cell-to-cell variability. The requirement for high resolution is fulfilled as each individual scattering pattern is determined by all structures within the illuminated area and we detect typical dimensions between 3.6 nm and 34.0 nm. At the same time, we achieve high throughput, as we examine roughly 800 cells, storing 8895234 scattering patterns in about 7 h. Furthermore, the vast amount of data provided by one acquisition can be dealt with thanks to the semi-automated segmentation of the corresponding dark-field contrast image.

Such segmentation enables three different kinds of analysis. First, the properties of the nuclear and cytoplasmic ROIs are analyzed as averages over the entire scanned area, thus characterizing the entire cell population. Second, a single cell is characterized *globally*: the nuclear and cytoplasmic scattering patterns belonging to the same cell are averaged, providing information about the ‘overall properties’ of that specific cell. To exemplify a statistically relevant analysis of a cell sub­population, we examine the dependence of quantitative structural parameters (*K* and α) on the cell size. Assuming the cell size to be indicative of the cell cycle phase, we find a decrease of *K* as the cell cycle proceeds from earlier phases (small cells) to later phases (large cells) of the cell cycle, thus supporting previous results (Hémonnot *et al.*, 2016*b* ▸) with data from many more cells, that is, 444 instead of 16. Third, a single scattering pattern analysis characterizes single cells *locally*: the scattering patterns are not averaged, so that the pseudo-resolution provided by the step size of the scan, 0.5 µm × 0.5 µm, is not lost.

The exposure time for a single scattering pattern is comparatively low (1.34 ms); however, this does not significantly impair the results of the power law fits with respect to those obtained using a longer exposure time, as shown by the comparison with a 20 ms exposure time scan, similar to what has been successfully used for cell scans in the past (Weinhausen *et al.*, 2014 ▸; Hémonnot *et al.*, 2016*a* ▸,*b* ▸). Importantly, while the radiation dose of the slower scans is comparable with values (Hémonnot & Köster, 2017 ▸) typical of scanning SAXS, *i.e.* about 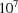
–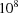
 Gy, with our method the dose is considerably lowered, ∼10^6^ Gy, comparable with ptychography. Overall, our approach lends itself to a variety of applications in the studies of subcellular structures and can be used as a high-throughput, label-free complementary method for other popular techniques such as fluorescence or electron microscopy.

## 5. Related literature   

The following reference, not cited in the main body of the paper, has been cited in the supporting information: Pearson (1901 ▸).

## Supplementary Material

Supporting text, figures and tables. DOI: 10.1107/S1600577520006864/ju5005sup1.pdf
