# Supplementary material for: Large field-of-view scanning small-angle X-ray scattering of mammalian cells
Source: J Synchrotron Radiat. 2020 Jun 16;27(Pt 4):1059–68. doi: 10.1107/S1600577520006864 (PMC7336178; doi:10.1107/S1600577520006864)
Supplement: Supplementary file 1 [file s-27-01059-sup1.pdf]

JOURNAL OF SYNCHROTRON RADIATION

Volume 27 (2020)

Supporting information for article:

Large field-of-view scanning small-angle X-ray scattering of mammalian cells

Chiara Cassini, Andrew Wittmeier, Gerrit Brehm, Manuela Denz, Manfred Burghammer and Sarah Köster

Detailed segmentation procedure

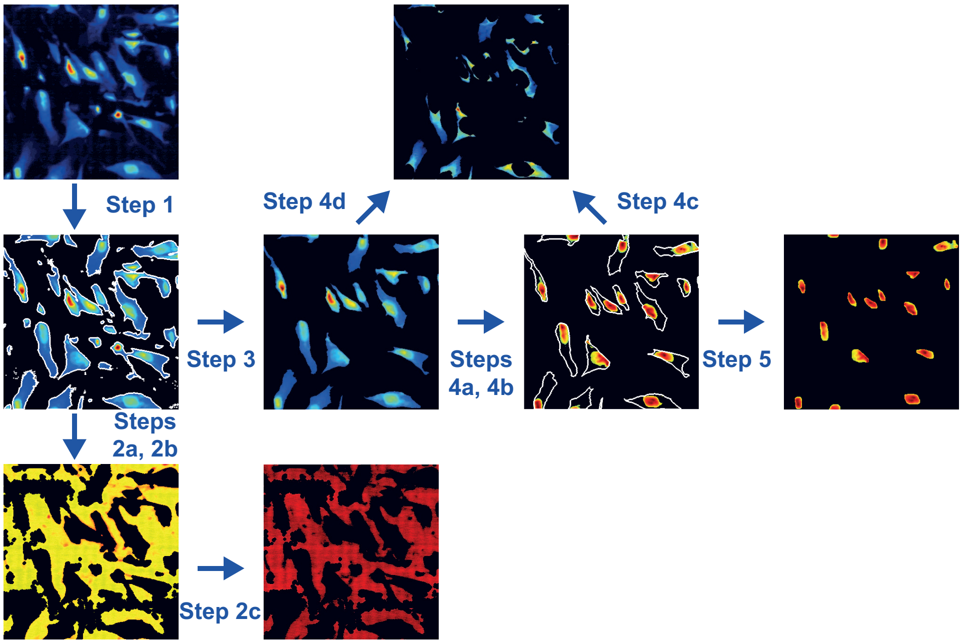

Figure S1: Schematic of the main steps of the segmentation procedure. The effects of the various steps are presented for a portion of the dark-field contrast image shown in Fig. 2a in the main text (the region inside the white box, also shown in Fig. 2b in the main text).

In the following, the complete procedure employed to obtain the segmentation presented in Fig. 2 in the main text is outlined. The results of the main steps of this procedure are summarized in Fig. S1.

1. **Local thresholding.** We first separate the background from the cells. Since the background is much darker (lower signal, fewer photons) than the cells, which are bright enough to be easily recognized by the human eye, a simple intensity threshold should in first approximation be sufficient. However, the background intensity is not constant throughout the dark-field contrast image, but varies, particularly along the vertical direction. This is caused by variations in the intensity of the incoming X-ray beam over the full duration of the scan of about 7 hours. Furthermore, there is a rather large variability in the intensity between different cells. As a result, some cells in the lower-intensity regions have parts of the cytoplasm that are roughly as intense as the background of the higher-intensity regions. For this reason, a single intensity value does not work as a threshold. A local thresholding strategy is more suitable. For each pixel of the dark-field contrast image, a local average intensity is computed within a $129\,\mathrm{px} \times 79\,\mathrm{px}$ box centered on the considered pixel itself. The pixel is regarded as

foreground if its intensity is above the local average, otherwise it is included in the background (Bradley's threshold (Bradley and Roth 2007)). The result is a logical mask with '1' at the locations of the foreground pixels and '0' at the positions of the background pixels.

2. **Background definition.** The first step in the background definition is a local thresholding step, where the threshold is 95% of the local average, so that a larger region than what was defined above is considered as foreground. The resulting mask then undergoes:

   (a) *inversion*, to switch from foreground to background;

   (b) *morphological closing* with a 4 px radius, disc-shaped structuring element, so that the contours of the region are smoothed and slightly expanded;

   (c) *intensity selection* on the background region, where only pixels with intensity values within one standard deviation from the average are retained.

3. **Cell bodies definition.** The foreground mask obtained in Step 1 is refined to yield single-cell contours through the following operations:

   (a) *morphological erosion* with a 2 px radius, diamond-shaped structuring element;

   (b) *size selection*, where only connected components containing a number of pixels equal to or greater than 250 are retained;

   (c) *manual cleaning*, where connected components are visually checked one by one and their contours are manually modified if necessary. Each of the final connected components must have exactly one nucleus.

4. **Cytoplasm definition.** In order to define the cytoplasmic region in each connected component, the cell bodies region obtained in the previous step undergoes:

   (a) cell by cell *contrast expansion*, where the intensity within each connected component is rescaled so that all available values are used. This step is just performed for better visualization and is not strictly necessary for the rest of the procedure to work;

   (b) cell by cell *global thresholding*, in which a nuclear mask is obtained for each connected component by selecting the pixels more intense than Otsu's threshold (Otsu 1979), calculated for the intensity distribution of the considered connected component.

   After this first raw nuclear region mask is obtained, it is

(c) *morphologically dilated* with a 4 px radius, diamond-shaped structuring element;

(d) *subtracted* from the cell bodies region mask. The resulting mask defines the cytoplasmic region.

5. **Nuclei definition.** Finally, the raw nuclear region mask obtained in Step 4b is refined by:

   (a) cell-by-cell *morphological opening* with a disk-shaped structuring element, with a surface area equal to 1/17 of the surface area of each cell's bounding box. This operation smooths the nuclear boundaries so that they assume a more disk-like shape;

   (b) *morphological erosion* with a 1 px radius, diamond-shaped structuring element;

   (c) *real nucleus selection*, where for each cell, only the biggest connected component is considered to be the nucleus, and other connected components in the cell body, if present, are rejected.

Isotropy of the 2D scattering patterns

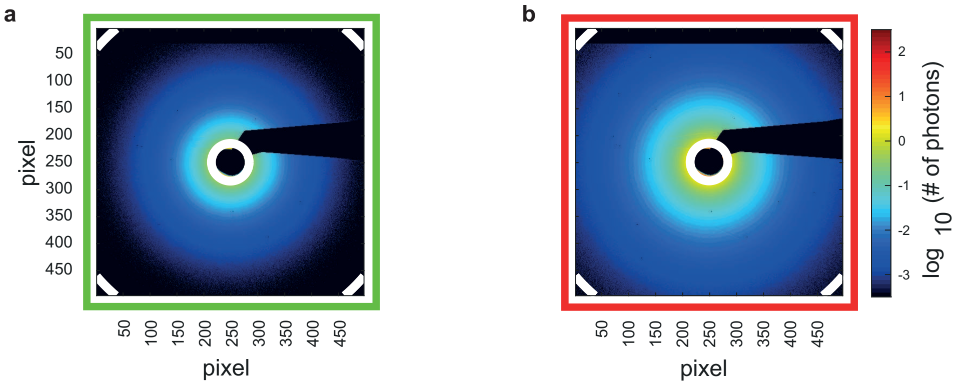

Figure S2: Isotropy of the average scattering patterns. a) Difference between the cytoplasmic average scattering pattern (Fig. 3b in the main text) and the background average scattering pattern (Fig. 3a in the main text). b) Difference between the nuclear average scattering pattern (Fig. 3c in the main text) and the background average scattering pattern (Fig. 3a in the main text). The white lines in a, b delimit the fitting range.

The isotropy of the average X-ray scattering signal from the cells clearly emerges if we carry out the background subtraction on the two-dimensional scattering patterns (Fig. S2) averaged over the entire window. This shows that the anisotropy visible in Fig. 3a, b and c in the main text are due to the setup only. Contributions may come from optics and collimation upstream of the sample and/or flight tube and beam stop downstream of the sample.

The background subtraction step is therefore essential for a correct data analysis. Local anisotropies of the sample are lost in the averaging process, but they can be present in the individual scattering patterns.

Selection of a subpopulation of cells

For the cell by cell analysis presented in the main text, only a subpopulation of the cells detected during image segmentation is considered. This is done mainly to reduce the computation time, but also to reduce the number of cellular fragments in the population. Indeed, our segmentation procedure cannot distinguish between a cell and a fragment of a cell, provided that the fragment is big enough and that it contains part of the nucleus. In our experience, fragmentation of the cells is not uncommon for freeze-dried samples such as ours. As a result, about 23% of the 912 cellular regions identified by our segmentation are really cellular fragments. By selecting only the cellular regions satisfying

- $N_{nuc} \geq 30$;
- $N_{nuc} \leq N_{cyt} \leq 5000$;
- $N_{cyt} < N_{bkg} \leq 10000$,

with N_{nuc}, N_{cyt} and N_{bkg} numbers of pixels included in the nuclear, cytoplasmic and background region of the given cellular region, respectively, we are left with 444 cellular regions, constituting the subpopulation of cells shown in Fig. 4b in the main text, of which only about 6% are fragments.

Artifacts of azimuthal integration

In order to obtain radial intensity profiles from the measured scattering patterns, an azimuthal integration needs to be performed. This means, in practice, that for each q value considered, a corresponding circumference is defined; subsequently, all the photon counts registered along that circumference are summed up; finally, this total number of counts is divided by the number of pixels along the circumference, so that the result is the intensity value $I(q)$ for the specific q value. The pixels corresponding to detector segments or to the beam stop are masked out (black pixels in Fig. S3a). Since these purely geometrical features are always the same throughout the scans (we use the same mask for all scans), for each q value the number of counts along a circumference is always divided by the same number, shown in Fig. S3d. Therefore, if we had only one photon count per q value, we would get just the reciprocal of the points in Fig. S3d, that is, the lowest black line in Fig. S3b, c, e and f. All the intensity values obtained as one count per circumference

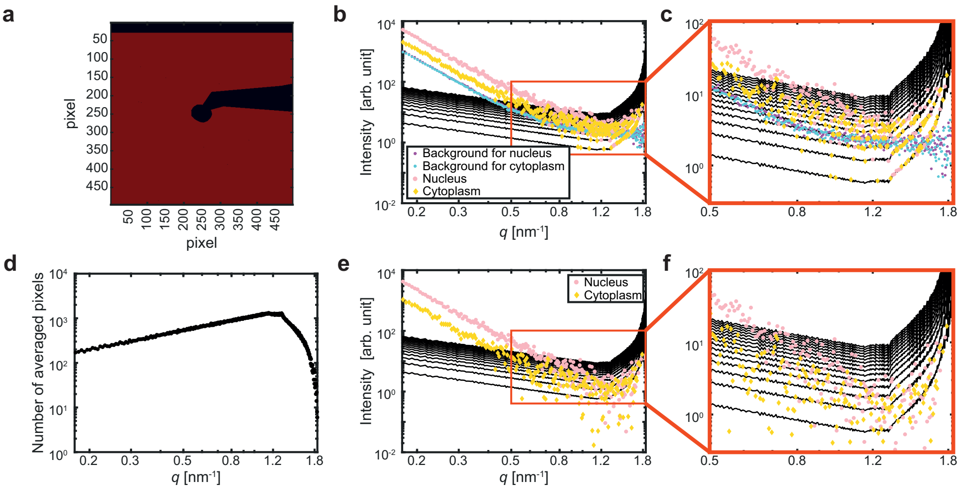

Figure S3: Artifacts due to the azimuthal integration of low-intensity scattering patterns. a) Logical mask defining which detector pixels are taken into account during the azimuthal integration procedure: red pixels are used, black pixels are ignored. b) Radial intensity profiles for the background (cyan for the cytoplasm, purple for the nucleus), for a cytoplasmic (yellow) and for a nuclear (pink) scattering pattern (the same ones as used in Fig. 4 in the main text). c) Enlargement of the region in the red box in b. d) Number of pixels whose value is averaged along a circumference corresponding to q, to obtain the intensity value $I(q)$. e) Background-subtracted radial intensity profiles for a cytoplasmic (yellow) and a nuclear (pink) scattering pattern (the same ones as used in Fig. 4 in the main text). f) Enlargement of the region in the red box in e. The black lines in b, c, e and f represent the intensity profiles we would get if we had only one photon count per q value (lowest line), only two (line just above the lowest) and so on up to 15 counts per q value (highest line). Note that the lowest black line is not exactly the reciprocal of the values in d, because it is normalized by the exposure time in order to match the data (also normalized). The other black lines have been normalized as well.

fall on this line; all the intensity values obtained as two counts per circumference fall on a line obtained multiplying the one-count line by two, and so on. The 15 black lines in Fig. S3b, c, e and f correspond to the values due to 1 to 15 photon counts per circumference. All intensity values lie on one of the lines that are multiples of the one-count line. This “discretization” effect, due to the fact that the possible radial intensity values are ratios of two integer numbers, is less visible for higher intensities, where the black lines would lie closer and closer together. The effect is reduced, or even eliminated, by averaging, as can be clearly seen in the background curves in Fig. S3b and c that are calculated from average scattering patterns. Since these background curves are much smoother because of the averaging, the background subtraction on the curves shown in Fig. S3b reduces this effect, as can be seen in Fig. S3e and f. However, the curves remain visibly noisy, displaying an increase in their terminal part due to the decrease of pixels associated with high q values (see Fig. S3d) as well as many extremely small intensity values (because the signal and the background differs only slightly)

or even missing points (when the signal is lower than the background, the difference is negative, so it can not be shown in a logarithmic plot).

Determination of the optimized exposure time

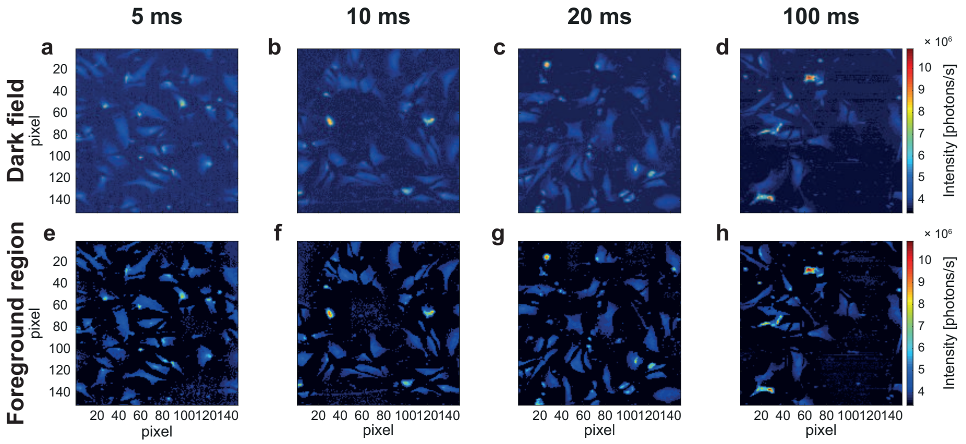

Figure S4: Dark-field contrast images obtained with different exposure times. a-d) Dark-field contrast images for exposure times of 5 ms, 10 ms, 20 ms and 100 ms per scan point, respectively; one pixel corresponds to 2 µm × 2 µm. e-h) Same as a-d, but the background region, obtained by local thresholding, is masked out. All images (a-h) are normalized by the exposure time and depicted on the same color scale.

Four 300 µm × 300 µm scans, each with the same step size (2 µm both horizontally and vertically) but with different exposure times (5 ms, 10 ms, 20 ms and 100 ms per scan point) are carried out in order to determine an optimized "long exposure time", used in the main text for comparison with the shorter exposure time of our window-sized scans (1.34 ms). The corresponding dark-field contrast images are shown in Fig. S4a-d. In order to evaluate the quality of the scans, the signal to noise ratio (SNR) of each dark-field contrast image is computed. The SNR is defined as

$$\mathrm{SNR} = \frac{I_{\mathrm{FRG}} - I_{\mathrm{BKG}}}{\sigma_{\mathrm{BKG}}}, \tag{S1}$$

where I_{FRG} is the average foreground intensity, I_{BKG} is the average background intensity and σ_{BKG} is the standard deviation of the background intensity. The foreground is determined with a local threshold, in analogy to the first step of the segmentation procedure illustrated above; in this case, the local average intensity is calculated within a 35 px × 35 px box. Since only an estimate of the foreground region is needed here, the foreground mask is not further processed; the results are shown in Fig. S4e-h. All the pixels of the dark-field contrast images that do not belong to the foreground are considered background. According to the resulting SNR values, presented in

Table S1, the best (highest) value corresponds to an exposure time of 20 ms.

Table S1: SNR for the dark-field contrast images shown in Fig. S4a-d.

Exposure time [ms]	SNR
5	7.8
10	9.8
20	10.4
100	8.4

Orientation and anisotropy of single scattering patterns

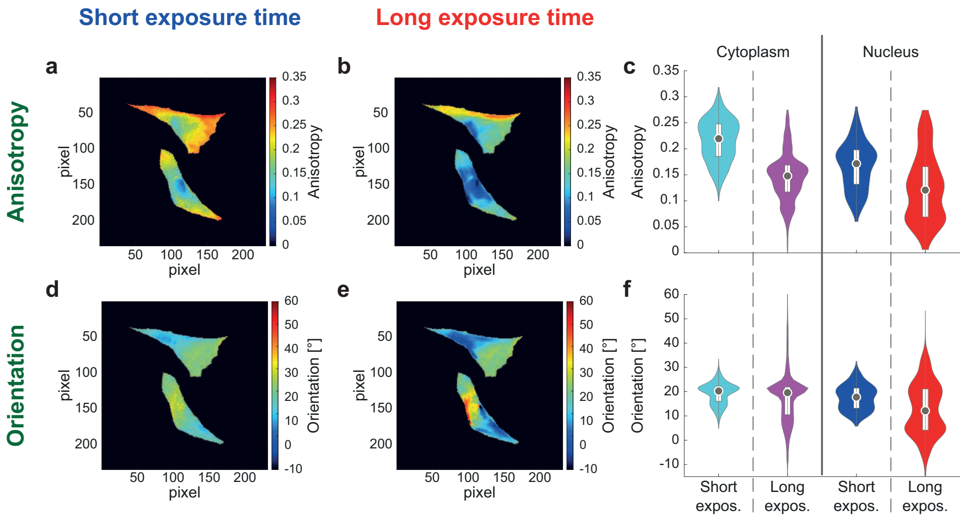

Figure S5: Comparison of anisotropy analysis for the fast and slow scans examined in the main text (Fig. 5). a) Map of anisotropy values, fast scan. b) Map of anisotropy values, slow scan. c) Violin plots of the anisotropy values shown in a, b. d) Map of orientation values, fast scan. e) Map of orientation values, slow scan. f) Violin plots of the orientation values shown in d, e. In the violin plots, the gray circles mark the median value and the white boxes represent the interquartile range.

In order to quantify the local anisotropy and orientation of the scanned points of the sample, a principal component analysis (PCA) (Pearson 1901) is performed on individual scattering patterns. The complete PCA procedure is explained in detail in Refs. (Bernhardt et al. 2016; Nicolas et al. 2017). Briefly, the covariance matrix of the scattering vector in the detector plane is diagonalized. The eigenvector corresponding to the larger eigenvalue defines the orientation of the scattering pattern. The corresponding orientation in real space is perpendicular to this direction. The anisotropy is defined as

the absolute value of the difference between the two eigenvalues, divided by their sum. The PCA is performed in the q range $[0.1744, 0.7664]\,\mathrm{nm}^{-1}$, corresponding to real space features between 8.2 nm and 36.0 nm. The results are shown in Figure S5.

Additional tables

Table S2: K and α obtained from the different data ensembles illustrated in Fig. 4 in the main text. Note that for the entire window and single scattering pattern analysis, the fit coefficients K and α are given, with the standard error computed from the fit in parentheses. For the cell by cell analysis, instead, the average value for each distribution is given, with the standard deviation of the distribution in parentheses.

	Entire window Fit value (Std. err.)	Cell by cell Average (Std. dev.)	Single scattering pattern Fit value (Std. err.)
K, cytoplasm [arb. unit]	0.468 (0.004)	0.48 (0.16)	0.56 (0.07)
K, nucleus [arb. unit]	1.71 (0.02)	1.7 (0.6)	2.08 (0.11)
α, cytoplasm	−4.265 (0.007)	−4.26 (0.04)	−4.36 (0.10)
α, nucleus	−4.277 (0.009)	−4.27 (0.03)	−4.33 (0.04)

Table S3: Selected statistical descriptors (median, standard deviation, average and standard error of the average) of the distributions shown in Fig. 5e and h in the main text. The cytoplasmic region consists of of 3394 data points, the nuclear region of 1393 data points. Values obtained with the short exposure time (1.34 ms) are shown in blue, values obtained with the long exposure time (20 ms) are shown in red.

	Exposure	Median	Standard deviation	Average (Std. err.)
K, cytoplasm [arb. unit]	short	0.470	0.224	0.519 (0.004)
	long	0.428	0.234	0.493 (0.004)
K, nucleus [arb. unit]	short	1.401	0.320	1.420 (0.009)
	long	1.476	0.366	1.461 (0.010)
α, cytoplasm	short	−4.235	0.129	−4.244 (0.002)
	long	−4.1841	0.0748	−4.1785 (0.0013)
α, nucleus	short	−4.2610	0.0548	−4.2610 (0.0015)
	long	−4.1898	0.0268	−4.1906 (0.0007)

Table S4: Average values of the distributions of K values (Fig. 6d, g in the main text) and of α values (Fig. 6c, h in the main text) for small and large cells. The standard deviation of each distribution is given in parentheses. Note that the standard deviation of the distribution is preferred here to the standard deviation of the mean, in order to highlight the properties of the complete distribution, instead of focusing on the average value only.

	Small cells Average (Std. dev.)	Large cells Average (Std. dev.)
K, cytoplasm [arb. unit]	0.59 (0.17)	0.38 (0.11)
K, nucleus [arb. unit]	2.2 (0.6)	1.3 (0.3)
α, cytoplasm	−4.27 (0.04)	−4.25 (0.03)
α, nucleus	−4.28 (0.03)	−4.26 (0.03)

References

Bernhardt, Marten et al. (2016). "X-Ray Micro- and Nanodiffraction Imaging on Human Mesenchymal Stem Cells and Differentiated Cells". In: *Biophys. J.* 110.3, pp. 680–690. DOI: 10.1016/j.bpj.2015.12.017.

Bradley, Derek and Gerhard Roth (2007). "Adaptive Thresholding Using the Integral Image". In: *J. Graph. Tools* 12.2, pp. 13–21. DOI: 10.1080/2151237x.2007.10129236.

Nicolas, Jan-David et al. (2017). "Scanning X-Ray Diffraction on Cardiac Tissue: Automatized Data Analysis and Processing". In: *J. Synchrotron Radiat.* 24.6, pp. 1163–1172. DOI: 10.1107/s1600577517011936.

Otsu, Nobuyuki (1979). "A Threshold Selection Method from Gray-Level Histograms". In: *IEEE Transactions on Systems, Man, and Cybernetics* 9.1, pp. 62–66. DOI: 10.1109/tsmc.1979.4310076.

Pearson, Karl (1901). "LIII. On lines and planes of closest fit to systems of points in space". In: *Philos. Mag.* 2.11, pp. 559–572. DOI: 10.1080/14786440109462720.
